# Supplementary material for: Alleviation of Hyperuricemia by Strictinin in AML12 Mouse Hepatocytes Treated with Xanthine and in Mice Treated with Potassium Oxonate
Source: Biology (Basel). 2023 Feb 17;12(2):329. doi: 10.3390/biology12020329 (PMC9953564; doi:10.3390/biology12020329)
Supplement: Supplementary file 1 [file biology-12-00329-s001.zip › biology-2183236-supplementary.pdf]

Supplementary Materials

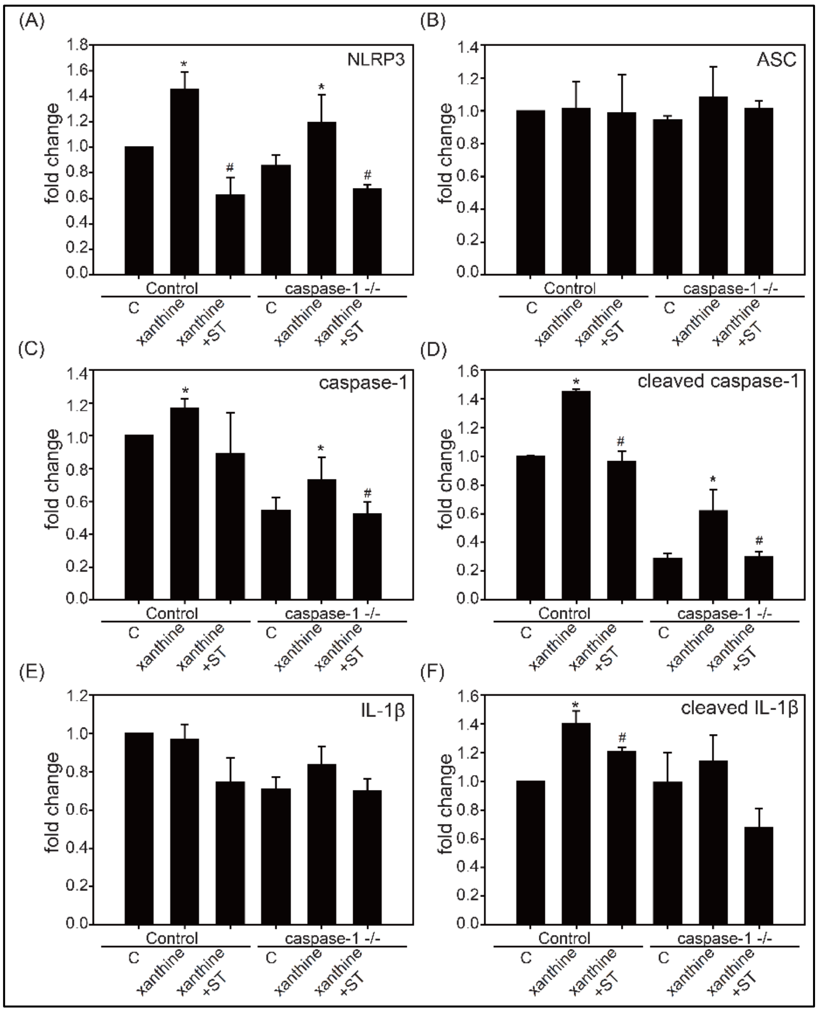

Figure S1. The quantitation of the Western blot showed protein expressions related to (A) NLRP3, (B) ACS, (C) caspase-1, (D) cleaved caspase-1, (E) IL-1β, and (F) cleaved IL-1β was shown in AML12 control and caspase-1 silenced cells (caspa-se-1 -/-) treated with xanthine alone or combined with ST for 24 hrs. Data were presented as mean ± SD from three independent experiments.*p<0.05 compared with control. #p<0.05 compared with xanthine alone groups.

Supplementary Materials, Fig. S2

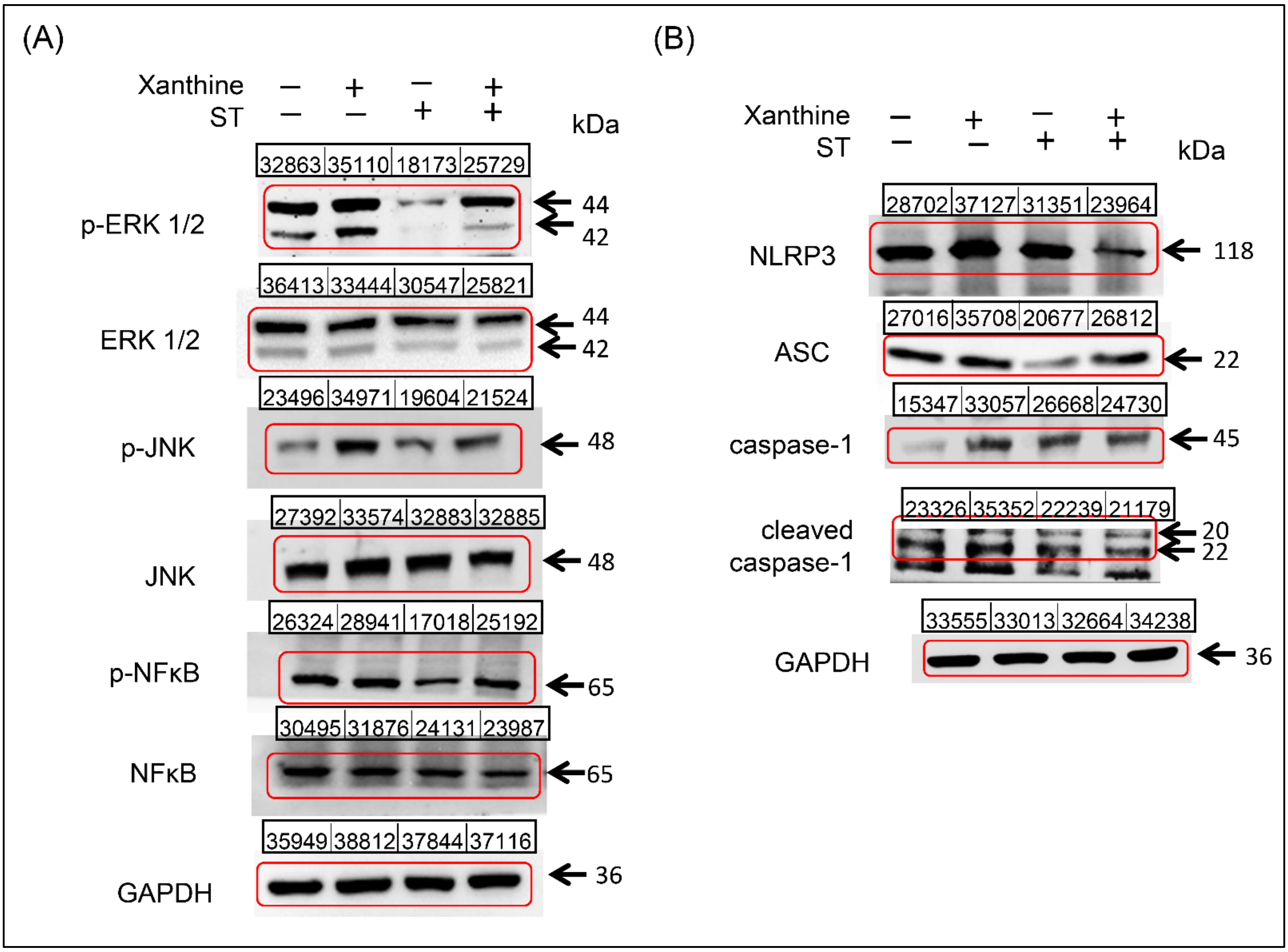

Figure S2. Western blot membrane of the representative data and densitometry readings are shown in Figure S2A and B. Molecular weight of proteins were shown as arrow indicated. Primary antibodies against NLRP3 (ab270449), caspase-1 (ab138483), and ASC (ab175449) were purchased from Abcam (Cambridge, MA, USA). Primary antibodies against JNK (2155-1) were purchased from Epitomics (Cam-bridge, MA, USA). Primary antibody against IL-1β (200-01B) was purchased from PeproTech (Rocky Hill, NJ, USA). Primary antibodies against GAPDH (D4C6R), p-NF-κB (#3039), NF-κB (#3034), p-ERK1/2 (#9911), ERK1/2 (#9102), and p-JNK (#9251) as well as horseradish peroxidase-conjugated anti-mouse (#7076) and anti-rabbit (#7074) secondary antibodies were purchased from Cell Signaling Technology (Beverly, MA, USA). The results of Western blot was analyzed using the enhanced chemiluminescent reagent (Invitrogen) and quantitated by the iBright Imaging Systems (iBright FL 1000; Thermo Fisher Scientific, Inc., Waltham, MA, USA), therefore the protein marker is not shown. In addition, the PVDF membranes are cut into small pieces to detect different proteins with different molecular weight.

Supplementary Materials, Fig. S3

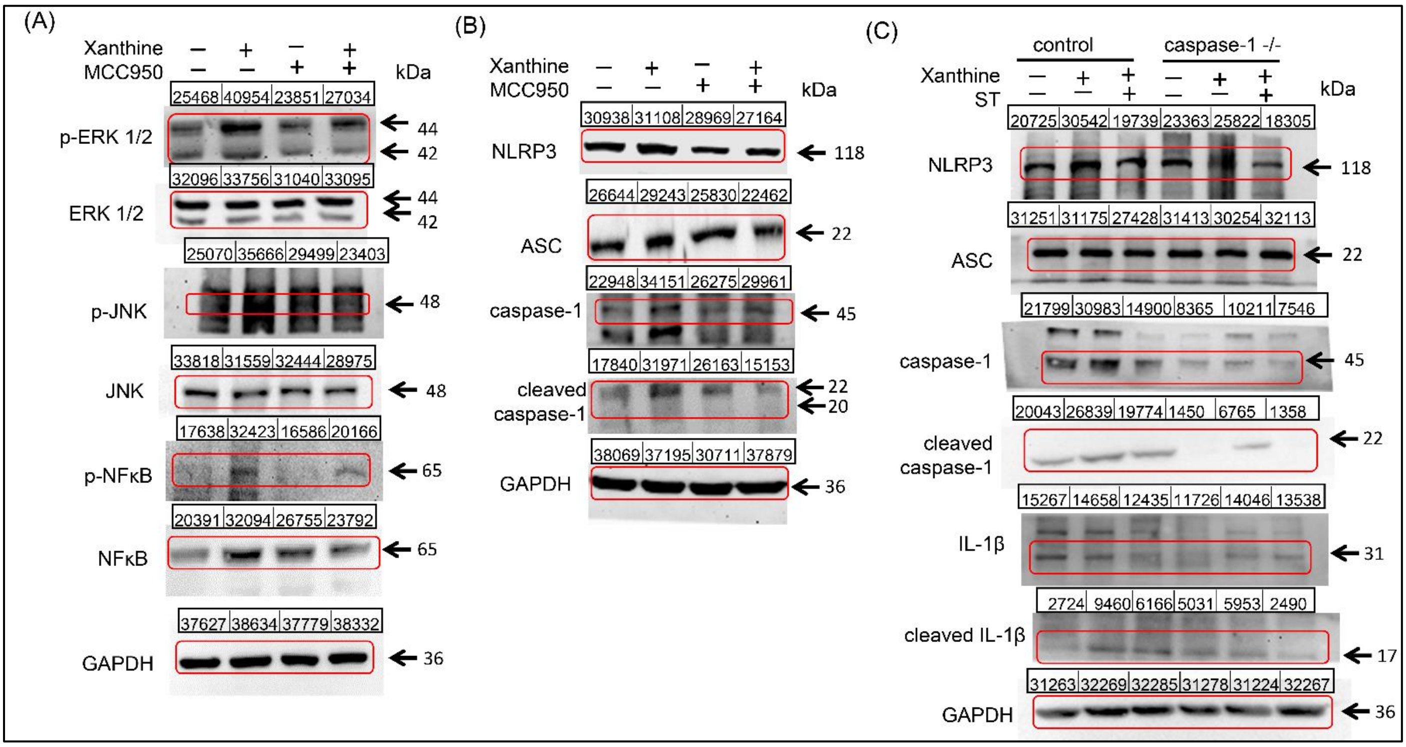

Figure S3. Western blot membrane of the representative data and densitometry readings are shown in Figure S3A, B, and 3C. Molecular weight of proteins were shown as arrow indicated. Primary antibodies against NLRP3 (ab270449), caspase-1 (ab138483), and ASC (ab175449) were purchased from Abcam (Cambridge, MA, USA). Primary antibodies against JNK (2155-1) were purchased from Epitomics (Cam-bridge, MA, USA). Primary antibody against IL-1β (200-01B) was purchased from PeproTech (Rocky Hill, NJ, USA). Primary antibodies against GAPDH (D4C6R), p-NF-κB (#3039), NF-κB (#3034), p-ERK1/2 (#9911), ERK1/2 (#9102), and p-JNK (#9251) as well as horseradish peroxidase-conjugated anti-mouse (#7076) and anti-rabbit (#7074) secondary antibodies were purchased from Cell Signaling Technology (Beverly, MA, USA). The results of Western blot was analyzed using the enhanced chemiluminescent reagent (Invitrogen) and quantitated by the iBright Imaging Systems (iBright FL 1000; Thermo Fisher Scientific, Inc., Waltham, MA, USA), therefore the protein marker is not shown. In addition, the PVDF membranes are cut into small pieces to detect different proteins with different molecular weight.
